# How do treadmill speed and terrain visibility influence neuromuscular control of guinea fowl locomotion?

**DOI:** 10.1242/jeb.104646

**Published:** 2015-10

**Authors:** Joanne C. Gordon, Jeffery W. Rankin, Monica A. Daley

**Affiliations:** Structure and Motion Laboratory, Royal Veterinary College, Hawkshead Lane, Hatfield, Hertfordshire AL9 7TA, UK

**Keywords:** Bipedal, Bird, Visuomotor control, Stability, *Numida meleagris*, Muscle

## Abstract

Locomotor control mechanisms must flexibly adapt to both anticipated and unexpected terrain changes to maintain movement and avoid a fall. Recent studies revealed that ground birds alter movement in advance of overground obstacles, but not treadmill obstacles, suggesting context-dependent shifts in the use of anticipatory control. We hypothesized that differences between overground and treadmill obstacle negotiation relate to differences in visual sensory information, which influence the ability to execute anticipatory manoeuvres. We explored two possible explanations: (1) previous treadmill obstacles may have been visually imperceptible, as they were low contrast to the tread, and (2) treadmill obstacles are visible for a shorter time compared with runway obstacles, limiting time available for visuomotor adjustments. To investigate these factors, we measured electromyographic activity in eight hindlimb muscles of the guinea fowl (*Numida meleagris*, *N*=6) during treadmill locomotion at two speeds (0.7 and 1.3 m s^−1^) and three terrain conditions at each speed: (i) level, (ii) repeated 5 cm low-contrast obstacles (<10% contrast, black/black), and (iii) repeated 5 cm high-contrast obstacles (>90% contrast, black/white). We hypothesized that anticipatory changes in muscle activity would be higher for (1) high-contrast obstacles and (2) the slower treadmill speed, when obstacle viewing time is longer. We found that treadmill speed significantly influenced obstacle negotiation strategy, but obstacle contrast did not. At the slower speed, we observed earlier and larger anticipatory increases in muscle activity and shifts in kinematic timing. We discuss possible visuomotor explanations for the observed context-dependent use of anticipatory strategies.

## Abstract (precis)

**Summary:** Guinea fowl (*Numida meleagris*) show speed-dependent shifts in neuromuscular control during obstacle negotiation, characterized by a greater reliance on anticipatory modulation and stride-to-stride neural adjustments at slow speed, shifting towards feedforward activation and intrinsic mechanical stability at high speed.

## INTRODUCTION

Legged animals navigate complex terrain by flexibly integrating multiple sensory modalities in a hierarchically organized control system that includes short-latency spinal reflexes, rhythmic spinal networks and higher central input via the motor cortex and descending pathways (Dickinson et al., 2000; Pearson, 2000; Nishikawa et al., 2007; Prochazka and Ellaway, 2012). To manoeuvre through changing terrain, motor control must be adapted through both anticipatory and reactionary mechanisms (Yakovenko et al., 2005; Marigold and Patla, 2007; Donelan et al., 2009). Here, we define anticipatory control as motor output generated prior to a goal-directed movement, originating from higher brain centres based on an internal predictive model and transmitted via descending spinal pathways (Pearson, 2000; Yakovenko et al., 2004; Frigon and Rossignol, 2006). Sensory information does feed into anticipatory control, but primarily acts upstream of spinal networks in the selection, planning and initiation of behaviour in the higher central nervous system (Patla, 1998; Marigold and Patla, 2007; Fajen et al., 2013).

In contrast, we define reactionary control as feedback modulation of motor output following limb contact with the terrain, resulting from deviations between anticipated and actual dynamics, predominantly coordinated via short-latency reflex feedback to spinal networks (Moritz and Farley, 2005; Daley et al., 2009). The reactionary response to a perturbation represents a combination of intrinsic limb dynamics in response to terrain contact, and sensory feedback relaying the errors between anticipated and perceived body states (Zehr and Stein, 1999; Moritz and Farley, 2004; Ross and Nichols, 2009). Thus, an interplay occurs between anticipatory and reactionary control mechanisms, because reactionary responses depend on the extent to which anticipatory control has adjusted limb trajectory, posture and impedance appropriately in advance of contact with terrain.

When adequate sensory information is available, anticipatory control should allow animals to optimize movement for the task and environment, to facilitate economic and robust locomotion (Cinelli and Patla, 2008; da Silva et al., 2011; Matthis and Fajen, 2014). We expect animals to use anticipation when possible, because accurate forward planning may improve foot and limb positioning, which could maximize stability and minimize energy cost (Matthis and Fajen, 2013). When terrain is uneven and unpredictable, gait is more variable and energy costs increase markedly (Voloshina et al., 2013). Anticipatory control of limb trajectory may allow animals to move in ways that minimize muscle work and force, minimizing muscular effort, as recently suggested by simulation studies (Birn-Jeffery et al., 2014; Van Why et al., 2014). Nonetheless, reactionary mechanisms are likely to be of similar importance in locomotion, buffering against unexpected perturbations when visual information is insufficient to accurately anticipate terrain conditions (Moritz and Farley, 2004; Daley and Biewener, 2011).

Recent research suggests ground birds vary their use of anticipatory strategies when negotiating uneven terrain between overground and treadmill conditions. Birds negotiating obstacles overground use anticipatory manoeuvres to vault upwards onto obstacles to avoid excessively crouched postures on the obstacle (Birn-Jeffery, 2012; Birn-Jeffery and Daley, 2012; Birn-Jeffery et al., 2014). In contrast, guinea fowl running over camouflaged obstacles on a treadmill do not use anticipatory strategies, exhibiting pre-obstacle dynamics similar to those of level terrain (Daley and Biewener, 2011). Therefore, birds land with a crouched posture on obstacles, subsequently recovering through posture-dependent changes to muscle force and work (Daley and Biewener, 2011). Given that guinea fowl use anticipatory strategies for overground obstacles, even at high running speeds (4 m s^−1^ and higher) (Birn-Jeffery, 2012; Birn-Jeffery and Daley, 2012; Birn-Jeffery et al., 2014), it is unclear why anticipatory strategies were not used in treadmill obstacle negotiation, especially considering the repetitive nature of terrain presentation.

**List of symbols and abbreviations**

AIC: Akaike information criterion
CL: contralateral footfall stride sequence
*E*_tot_: total myoelectric intensity
EMG: electromyographic
FCLP: flexor cruris lateralis pelvica
FPPD3: flexores perforati digiti III
FTL: femorotibialis lateralis
IC: iliotibialis cranialis
IF: iliofibularis lateralis
ILPO: iliotibialis lateralis postacetabularis
LG: lateral gastrocnemius
MG: medial gastrocnemius
PC: principal component
PCA: principal component analysis
S: obstacle stride footfall sequence

We hypothesized that the difference in obstacle negotiation strategies between overground and treadmill settings relates to differences in visual sensory information, which influence the ability to effectively execute anticipatory manoeuvres. While multiple sensory, dynamic and neuromuscular factors could explain the context-dependent use of anticipatory control (Müller et al., 2010; Belmonti et al., 2013; Matthis and Fajen, 2013; Birn-Jeffery et al., 2014), vision is the most prominent sensory difference between the experiments described above. Human studies have demonstrated that visual perception plays a central role in safe and efficient anticipatory route planning through complex terrain (Hollands et al., 2002; Patla et al., 2002; Mohagheghi et al., 2004; Marigold and Patla, 2008; Matthis and Fajen, 2013). Overground conditions may provide greater visual information than treadmill conditions because: (1) optical flow is reduced on a treadmill, reducing the visual stimulus compared with overground conditions, (2) obstacles are visible for a longer approach distance in overground locomotion, allowing a longer viewing time for anticipatory visuomotor adjustments, and (3) treadmill obstacles may have been visually imperceptible as a result of low contrast to the substrate (<10% contrast) and uniform lighting, particularly in the study design of Daley and Biewener (2011).

On treadmills, the short time between obstacle appearance on the belt and obstacle encounter, which is approximately one stride, may restrict anticipatory control because of visuomotor latencies. In humans, the time available for terrain assessment and visuomotor response is a critical factor in the use of anticipatory strategies (Patla, 1997; Patla and Vickers, 2003). Time available for visual assessment of terrain specifically influences steering, path planning and foot-placement behaviour during walking in cats and humans (Patla, 1997; Fowler and Sherk, 2003). Walking humans target gaze two steps ahead, creating a minimum visuomotor response time of two step periods, which allows them to maintain speed and stability compared with conditions with limited vision (Patla, 1997; Marigold and Patla, 2007; Matthis and Fajen, 2013). Gaze distance and minimal visuomotor response time have not been studied in birds, to our knowledge. However, treadmills can restrict terrain viewing time to one step period or less, by restricting gaze to the short length of the treadmill belt. If birds, like humans and cats, normally gaze two steps ahead, visually mediated anticipatory control may be particularly restricted at higher treadmill speeds, when the terrain appears a very short time before it is encountered.

The goal of this study was to investigate the effects of (1) terrain visibility (high/low contrast) and (2) treadmill speed on the neuromuscular control strategies used by guinea fowl during obstacle negotiation. We recorded obstacle negotiation at two treadmill speeds, 0.7 and 1.3 m s^−1^, with the obstacle at the same fixed distance at both speeds. At the slower speed, the bird has a longer time between obstacle appearance at the front of the treadmill belt and its encounter in the middle of the treadmill. Additionally, we manipulated the strength of the terrain visual stimulus by using two different obstacle conditions: low-contrast obstacles (<10% contrast, black/black) and high-contrast obstacles (>90% contrast, black/white). These conditions were selected to investigate whether low obstacle visibility or speed effects on obstacle viewing time contributed to the lack of observed anticipatory control strategies in the previous study of guinea fowl negotiating treadmill obstacles (Daley and Biewener, 2011). We used a treadmill experimental setup because the running speed of animals cannot be easily controlled in overground settings.

We hypothesized that the use of anticipatory control strategies for obstacle negotiation would be greater (1) for high-contrast obstacles and (2) at the slower treadmill speed, when obstacle viewing times are longer. Anticipatory control should manifest as larger shifts in muscle activity and gait kinematics in strides preceding foot contact with the obstacle, when compared with level-terrain locomotion at the same speed. At higher speeds, obstacles will be visible on the belt for a shorter time before their encounter, which may restrict visuomotor responses. We therefore expected the greatest anticipatory effects to be observed during trials with high-contrast obstacles and slower speed. If we observed no anticipatory control for treadmill obstacle negotiation across all measured conditions, this would suggest a fundamental difference in neuromuscular control between treadmill and overground locomotion, possibly due to sensory differences such as reduced optical flow.

## RESULTS

**Fig. 1.**
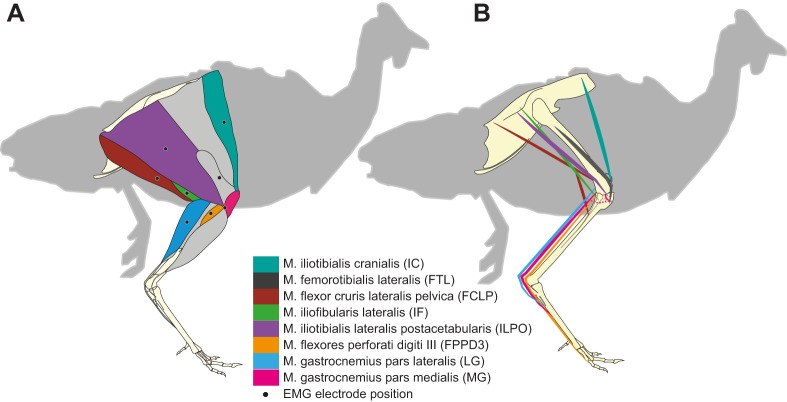
**Schematic diagram of guinea fowl hindlimb anatomy and EMG electrode placement.** (A) Muscle anatomy and placement of the eight electrodes. (B) Skeletal anatomy with each muscle's line of action to illustrate origin and insertion. The dashed line represents the medial section of the proximal head of the medial gastrocnemius (MG).

**Fig. 2.**
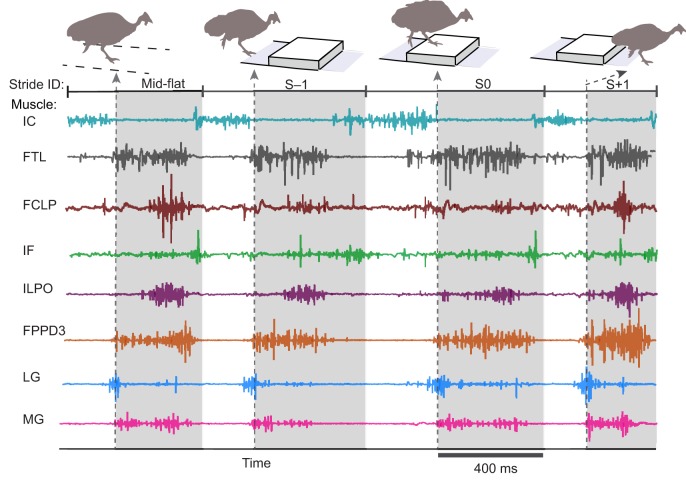
**A representative four-stride sequence of raw EMG data recorded from eight guinea fowl hindlimb muscles during treadmill obstacle negotiation in the high-contrast, slower speed condition.** Grey shaded regions indicate stance phase of the instrumented limb. Data are shown for one of two possible stride sequences (see Fig. 3) as the bird approaches, steps onto and steps over the obstacle. The recording limb underwent stance phase on top of the obstacle in stride ID ‘S0’.

**Fig. 3.**
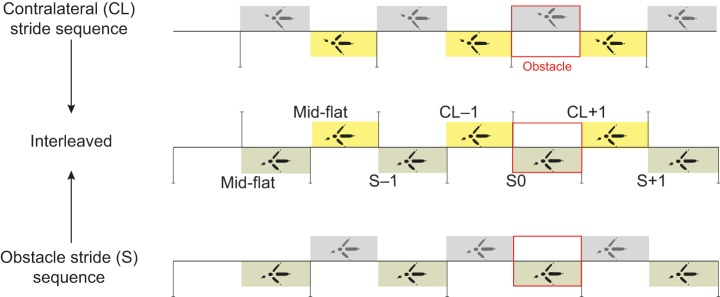
**Schematic representation of the two possible stride sequences of the instrumented right limb during obstacle negotiation, depicted from an ‘overhead’ foot step view.** Data cutting points are indicated by vertical black lines. The obstacle footfall event is outlined in red. The bottom panel depicts the obstacle stride footfall sequence (‘S’), in which the instrumented right leg enters a stance phase on the obstacle (S0), and the non-recording left leg undergoes stance directly before and after the obstacle. The top panel depicts the alternative contralateral footfall stride sequence (‘CL’), in which the instrumented right leg undergoes stance directly before (CL−1) and after (CL+1) the obstacle, whereas the non-recording leg enters stance on the obstacle. The middle panel shows these stride sequences interleaved, where instrumented limb data are used to produce a complete bilateral obstacle negotiation sequence, assuming symmetry between the right and left legs. Stride IDs shown in the middle panel are used in subsequent figures.

We report electromyographic (EMG) activity from eight hindlimb muscles spanning a proximo-distal distribution (Fig. 1, Table 1), recorded using indwelling electrodes (see Materials and methods). Trials were recorded for two speeds (0.7 and 1.3 m s^−1^) and three terrain conditions at each speed: (i) level, (ii) repeated 5 cm low-contrast obstacles (<10% contrast, black/black) and (iii) repeated 5 cm high-contrast obstacles (>90% contrast, black/white). We analysed strides between successive right limb toe-off events (Fig. 2), and categorized them in accordance with two possible obstacle negotiation sequences, classified by footfall relative to the obstacle (Fig. 3). In one sequence, the recording limb stepped onto the obstacle directly (S0), and the ipsilateral strides before and after obstacle contact were designated S−1 and S+1, respectively (Fig. 3, bottom, obstacle stride sequence ‘S’ of the right leg). This sequence corresponds to strides previously analysed by Daley and Biewener (2011). During this ‘obstacle stride’ sequence, the contralateral limb completes strides with stance events between S−1 and S0 immediately before the obstacle, and between S0 and S+1 just after the obstacle (Fig. 3, bottom, left leg sequence). We also recorded sequences in which the recording limb completed strides with stance events immediately before and after the obstacle, designated CL−1 and CL+1, with the contralateral (non-recording) limb encountering stance on the obstacle (Fig. 3, top, contralateral stride sequence). In the analysis and Fig. 3, we have interleaved the two sequences (Fig. 3, middle) to represent the entire bilateral obstacle negotiation pattern, assuming symmetry between the right and left legs.

**Table 1.**
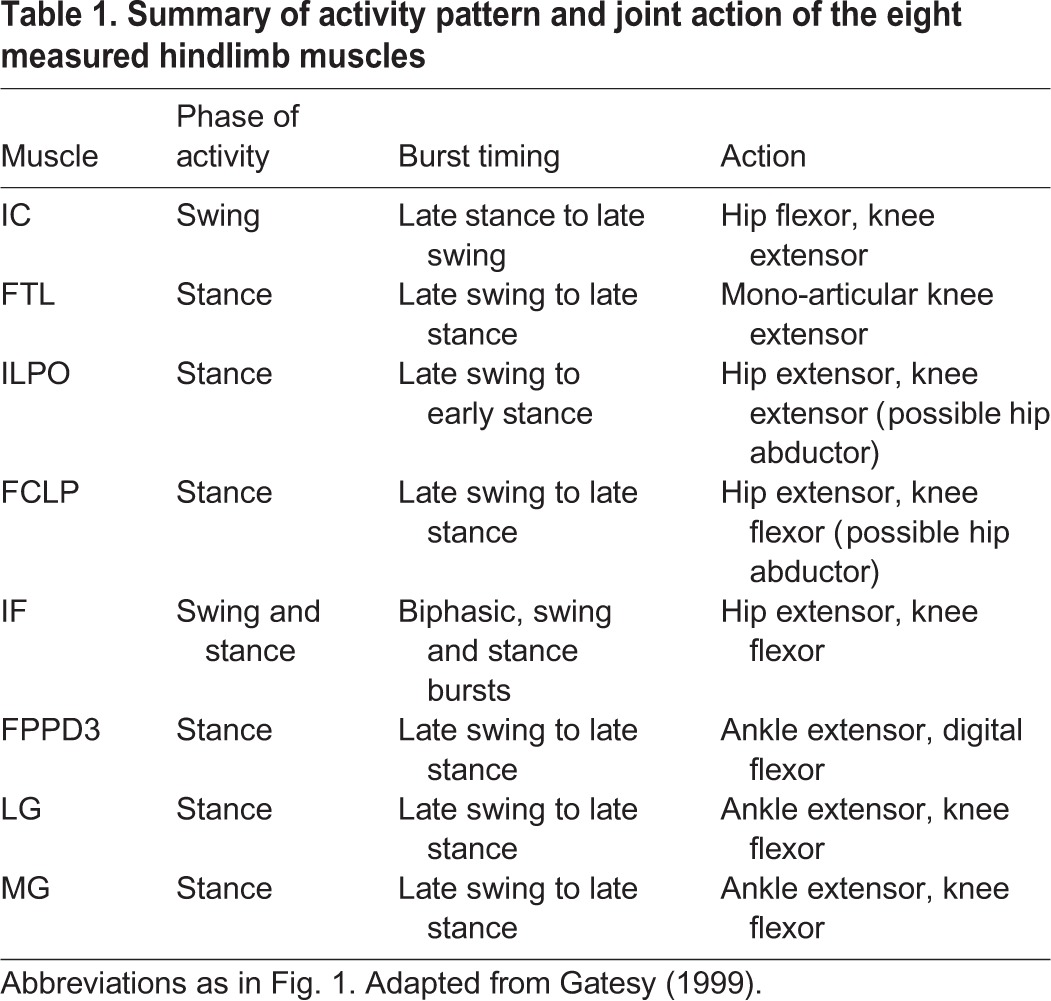
**Summary of activity pattern and joint action of the eight measured hindlimb muscles**

### Statistical summary

Several linear mixed effects models were evaluated in comparison to a reference model, to test for significant effects of speed and obstacle contrast on obstacle negotiation strategy (see Materials and methods). All models included individual as a random effect. The final model reported in Table 2 is that which resulted in the lowest Akaike information criterion (AIC) for all measured variables (Akaike, 1976), including total muscle activity per stride (*E*_tot_) and kinematic timing (see ‘Statistics’ in Materials and methods). The reported model includes the fixed effects ‘speed’, ‘stride ID’ and the interaction term ‘speed×stride ID’. The speed term quantifies generic speed effects, stride ID quantifies the obstacle negotiation strategy (across both speeds), and speed×stride ID quantifies speed-specific obstacle negotiation strategy. The factor stride ID has the largest explanatory power in the model (largest *F*-statistic, Table 2), suggesting that the overall shifts in muscle activity during obstacle negotiation are similar in magnitude between speeds. Nonetheless, across all muscles, the speed×stride ID interaction term contributed significant additional explanatory power to the model (Table 2), based on an *F*-statistic >1 and lower AIC compared with models without the term. The speed×stride ID term reflects speed-specific differences in the stride sequence during obstacle negotiation, suggesting shifts in neuromuscular strategy. In contrast, models including the fixed effect of ‘obstacle contrast’, either as a main effect or as an interaction term, did not improve the ANOVA model fit, as assessed by AIC. In summary, manipulating treadmill speed had a significant effect on muscle activity (*E*_tot_) and kinematic timing (Table 3), whereas obstacle contrast did not. Below, we report in detail the observed anticipatory and reactionary changes in hindlimb muscle activity and kinematic timing at the two treadmill speeds.

**Table 2.**
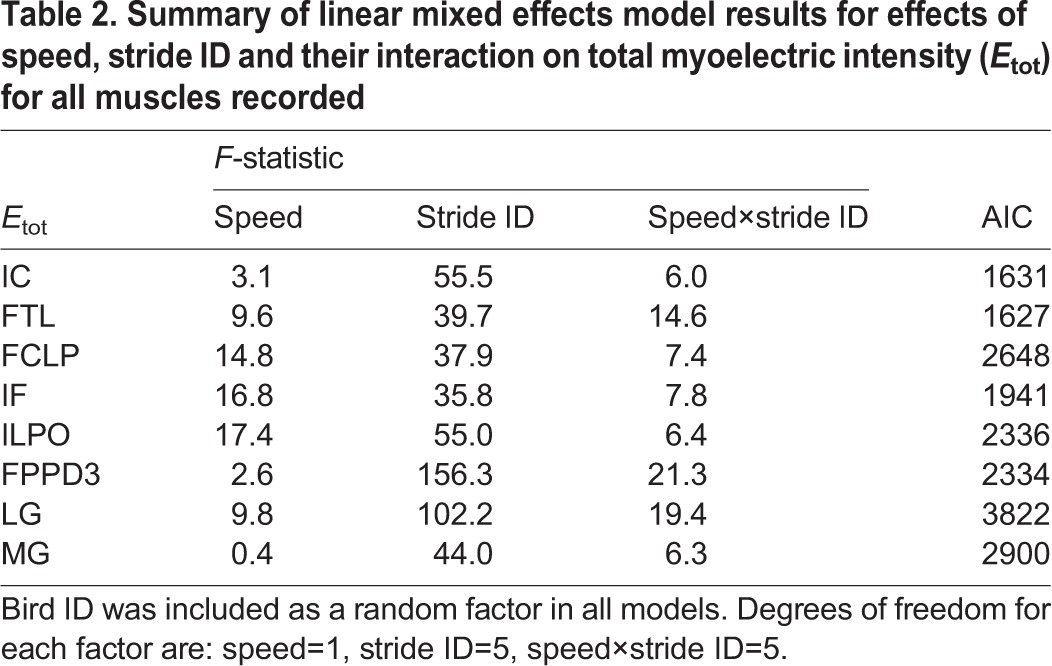
**Summary of linear mixed effects model results for effects of speed, stride ID and their interaction on total myoelectric intensity (*E*_tot_) for all muscles recorded**

**Table 3.**
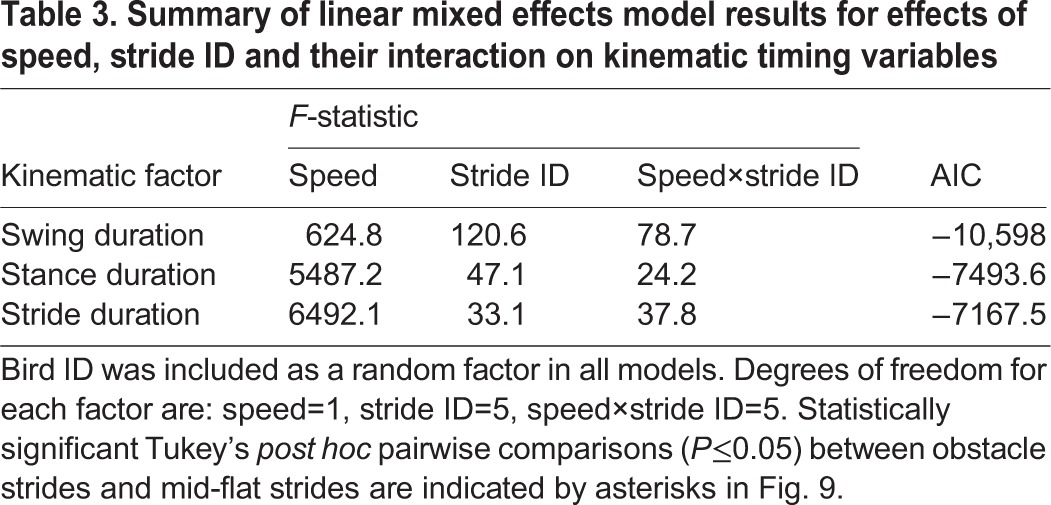
**Summary of linear mixed effects model results for effects of speed, stride ID and their interaction on kinematic timing variables**

### Anticipatory myoelectric intensity changes are larger at the slower speed

**Fig. 4.**
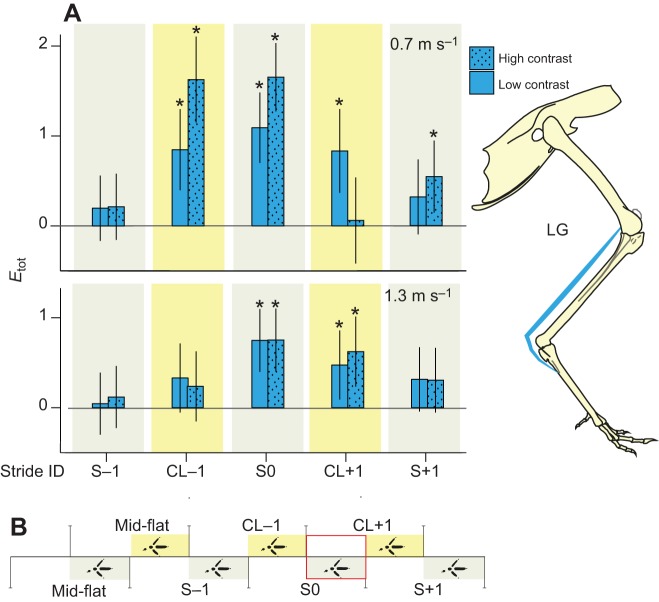
**Change in total myoelectric intensity per stride (*E*_tot_) during obstacle negotiation in the lateral gastrocnemius (LG), as a fractional difference from mid-flat strides.** (A) Changes in LG *E*_tot_ at the slower speed (top) and higher speed (bottom) for the bilateral obstacle negotiation sequence (B; see Fig. 3). Low- and high-contrast obstacle conditions in A are shown with solid and dotted bars, respectively. Bars indicate grand mean differences from mid-flat strides, with error bars indicating s.e.m. and asterisks for statistically significant *post hoc* pairwise differences from mid-flat strides (*P*≤0.05).

As lateral gastrocnemius (LG) activity during obstacle negotiation has been reported previously for a single speed (Daley and Biewener, 2011), we first highlight the general trends in this muscle across terrain conditions (Fig. 4). During slower speed obstacle negotiation (Fig. 4A, top), *E*_tot_ significantly increased in intensity before the obstacle encounter, in stride CL−1. Additionally, LG showed larger fractional increases in *E*_tot_ strides CL−1 and S0 when compared with the faster speed (Fig. 4A, bottom). Within a single speed and stride category, the high- and low-contrast conditions (Fig. 4A) did not exhibit statistically significant differences in *E*_tot_, based on Tukey’s *post hoc* pairwise comparisons.

**Fig. 5.**
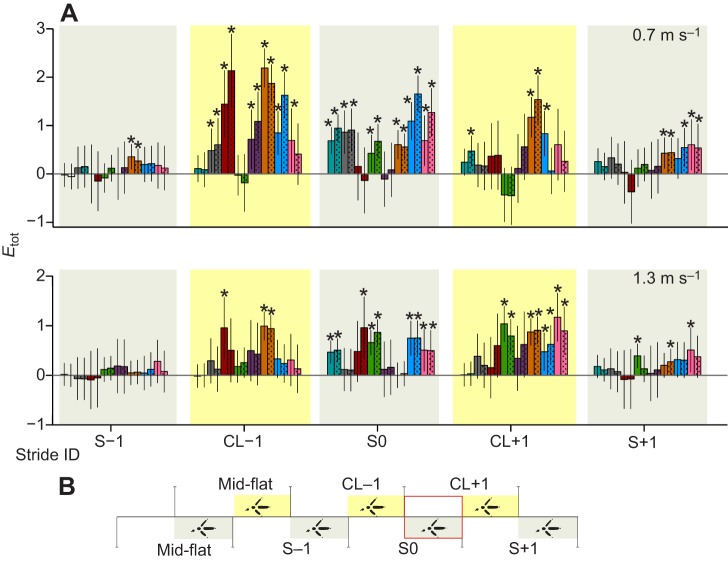
**Changes in *E*_tot_ during obstacle negotiation as a fractional difference from mid-flat strides, for all eight recorded hindlimb muscles.** (A) Changes in *E*_tot_ at the slower speed (top) and higher speed (bottom) for the bilateral obstacle negotiation sequence (B; see Fig. 3). Colour legend as in Fig. 1, with solid and dotted bars indicating low- and high-contrast obstacle conditions, respectively. Bars indicate grand mean differences in *E*_tot_ from mid-flat strides, with error bars indicating s.e.m. and asterisks showing statistically significant *post hoc* pairwise differences from mid-flat strides (*P*≤0.05).

When changes in total muscle recruitment, *E*_tot_, are examined across muscles (Fig. 5), the LG is representative of the overall trends. At the slower speed (Fig. 5A, top; supplementary material Table S1), numerous muscles showed significant increases in *E*_tot_ during strides preceding obstacle contact, including: flexores perforati digiti III (FPPD3) in stride S−1, and femorotibialis lateralis (FTL), flexor cruris lateralis pelvica (FCLP), iliotibialis lateralis postacetabularis (ILPO), FPPD3, LG and medial gastrocnemius (MG) in stride CL−1. In comparison, at the higher speed (Fig. 5A, bottom; supplementary material Table S2), only two muscles, FPPD3 and FCLP, showed a significant increase in *E*_tot_ preceding stride S0. Instead, muscles exhibited larger increases in *E*_tot_ during the CL+1 stride, following obstacle contact, with significant increases for iliofibularis lateralis (IF), FPPD3, LG and MG. We did not observe statistically significant differences between low- and high-contrast conditions, based on Tukey’s *post hoc* pairwise comparisons.

**Fig. 6.**
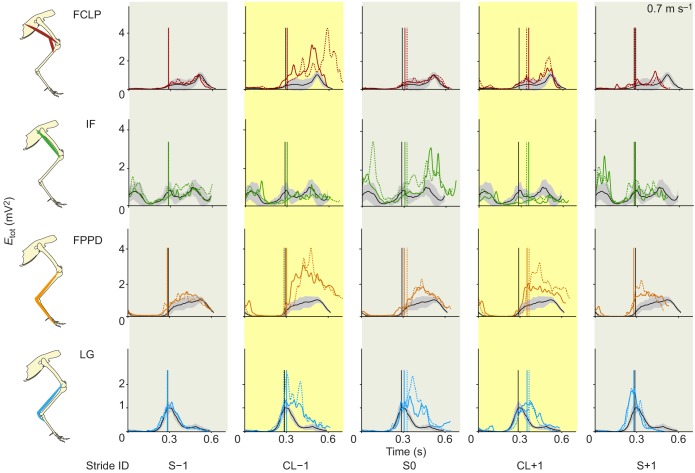
**Average trajectories of muscle activation during slower speed obstacle negotiation for four hindlimb muscles.** Stride sequence as shown in Fig. 3. Traces are grand means of *E*_tot_ as a function of time, shown for mid-flat strides (black with grey 95% confidence interval), low-contrast obstacle strides (solid coloured lines) and high-contrast obstacle strides (dashed coloured lines). Vertical lines indicate toe-down time (solid black for level, solid coloured for low-contrast obstacles and dashed coloured for high-contrast obstacles). We show four muscles here to represent the main patterns observed across the limb; see supplementary material Fig. S1 for the remaining muscles.

**Fig. 7.**
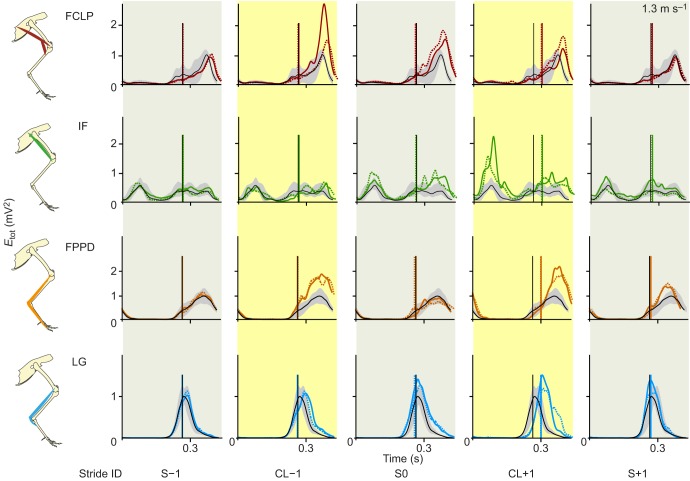
**Average trajectories of muscle activation during higher speed obstacle negotiation for four hindlimb muscles.** Colours and lines as in Fig. 6; see supplementary material Fig. S2 for the remaining muscles.

To further examine the detailed temporal changes in muscle activity during obstacle negotiation, we compared the stride-averaged myoelectric intensity trajectories between mid-flat and obstacle negotiation strides (Figs 6, 7; see supplementary material Figs S1, S2 for the remaining four muscles). In the stride preceding the obstacle (CL−1), the activity of FCLP, the multi-articular digital flexor FPPD3 and LG increased in magnitude at both speeds; however, the time course and duration of activity differed more at the lower speed (Figs 6, 7; supplementary material Fig. S1). In the obstacle stride (S0), the IF exhibited increased activity in both swing and stance phases of its double-bursting pattern, but again, changes in the time course and duration of activity were more pronounced for the slower speed (Figs 6, 7). LG increased activity during S0 at both speeds, but the slower speed showed a more pronounced shift in timing of peak activity toward later stance (Figs 6, 7). Across muscles, these trajectory plots revealed larger changes in the time course of activity at the slower speed (Fig. 6; supplementary material Fig. S1), whereas the characteristic shape of the activation pattern was relatively consistent at the faster speed (Fig. 7; supplementary material Fig. S2). Thus, the slower speed exhibited greater evidence of anticipatory changes in motor recruitment and stride timing during obstacle negotiation.

### Principal component analysis

We used principal component analysis (PCA) as a quantitative tool to examine the covariance of activation changes across all measured muscles and terrain conditions. The PCA revealed that the first two principal components (PCs) explain 75% of the variance in *E*_tot_ across all measured muscles and terrains (supplementary material Fig. S3). The first principal component (PC1) explained 53% of the variance and had high positive loadings for stance antigravity and leg extensor muscles (FTL, FCLP, ILPO, FPPD3, LG, MG), and one swing-active hip flexor that contributes to limb elevation (iliotibialis cranialis, IC). Thus, PC1 represents high covariance among seven of eight measured muscles (all except IF), and might therefore correspond to limb-wide co-activation for stance antigravity support and leg shortening/lengthening. IF did exhibit substantial stride-specific shifts in activity, but did not factor strongly in PC1, possibly due to its unusual double-bursting activation pattern (Figs 6, 7). The second principal component (PC2) explained 22% of the variance and had positive loadings for muscles that assist limb retraction (FCLP, ILPO, FPPD3), and negative loadings for a hip flexor that assists limb protraction (IC), and two multi-articular muscles that cross the knee (IF, MG). These loadings suggest PC2 might correspond to leg angular excursion (protraction/retraction). Overall, the PCA results demonstrate high covariance among hindlimb muscles, suggesting synergistic activation of muscles across the limb.

**Fig. 8.**
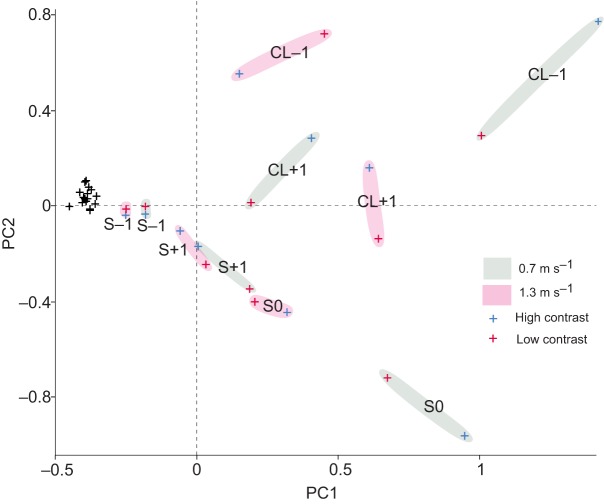
**Principal component analysis of variance in *E*_tot_ across eight hindlimb muscles and all measured terrain conditions.** Scores for the first two principal components (PC1 and PC2) explain 75% of the variance in *E*_tot_ across muscles, terrains and stride categories, indicating high covariance of limb muscle activity. Scores for PC1 are shown against those for PC2 for each stride category, with black ‘+’ for level terrain and mid-flat strides, blue ‘+’ for high-contrast obstacle strides and red ‘+’ for low-contrast obstacle strides. Shaded regions indicate clusters associated with speed and stride ID, illustrating speed-specific differences in obstacle negotiation strategy, but relatively lower variance associated with obstacle contrast. See Results for further details.

Covariance along PC1 and PC2 (Fig. 8) revealed distinct clusters associated with stride category and speed, with high- and low-contrast conditions grouping together. Slow-speed CL−1 pre-obstacle strides scored highest in both PC1 and PC2, whereas slow-speed S0 obstacle strides scored negatively in PC2 but positively in PC1. Positive scoring in both PC1 and PC2 (stride CL−1) is consistent with increased stance antigravity support and leg extension (+PC1) and increased leg retraction (+PC2). Positive scoring of PC1 with negative scoring in PC2 (stride S0) is consistent with reduced limb retraction (−PC2) with increased stance antigravity support and leg extension (+PC1). The high-speed, post-obstacle stride CL+1 scored moderately high in PC1, but near zero in PC2. Overall, these results suggest a greater anticipatory increase in muscle recruitment across many limb muscles at the slower speed (as shown by higher PC scores for CL−1 at the slower speed), with comparatively lower anticipatory changes and higher reactionary changes in recruitment at the higher speed (as shown by higher PC scores for CL+1 at the faster speed).

### Kinematics

**Fig. 9.**
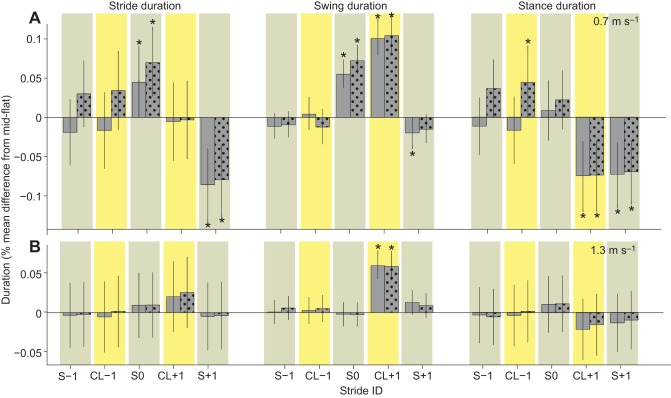
**Changes in kinematic timing: stride duration, swing duration and stance duration during obstacle negotiation.** Changes in duration are shown at the slower speed (A) and higher speed (B). Stride sequence as shown in Fig. 3, with solid and dotted bars for low- and high- contrast obstacles, respectively. Bars indicate the grand mean difference from mid-flat strides, with error bars indicating s.e.m. and asterisks for statistically significant *post hoc* pairwise differences from mid-flat strides (*P*<0.05).

Consistent with the muscle recruitment results, we observed larger stride-to-stride shifts in kinematic timing at the slower speed (Fig. 9A) compared with the higher speed (Fig. 9B). The ANOVA statistical results for kinematic timing variables revealed *F*-statistics >1 for the speed×stride ID interaction term (Table 3), suggesting significant differences in obstacle negotiation strategy between the two treadmill speeds. At the slower speed, swing period increased on the obstacle (S0) and when dismounting the obstacle (CL+1), and total stride duration also increased on the obstacle (S0). Stance duration decreased immediately following obstacle dismount (CL+1), and both stance and stride duration decreased in the subsequent post-obstacle stride (S+1). Higher contrast obstacles additionally resulted in increased stance duration preceding the obstacle (CL−1). Comparatively little stride-to-stride shifts in kinematic timing were evident during higher speed obstacle negotiation (Fig. 9B), with only a significantly prolonged swing period during the obstacle dismount (CL+1) compared with mid-flat strides.

## DISCUSSION

Our findings are consistent with the idea that a treadmill locomotion environment produces context-dependent shifts in sensorimotor control, possibly due to restricted visual information. Treadmills restrict visual information by (1) reducing optical flow, because only the treadmill belt is moving, (2) reducing obstacle contrast as a result of uniform terrain colour and lighting, and (3) reducing available obstacle viewing time, because of the sudden appearance of the obstacle at the front of the belt. These factors may reduce the quality of visual information and the time available for visuomotor modulation of motor output via descending pathways. In this study, we have manipulated (1) obstacle contrast and (2) treadmill speed to investigate the sensory factors that influence the use of anticipatory and reactionary neuromuscular control strategies. We hypothesized that use of anticipatory control strategies for obstacle negotiation would be greater (1) for high-contrast obstacles and (2) at the slower treadmill speed.

In contrast to our expectations for hypotheses 1, obstacle contrast did not significantly influence muscle recruitment patterns during obstacle negotiation, suggesting that it is not specifically contrast perception that influences the use of anticipatory strategies in this experimental context. Instead, we found that guinea fowl can use anticipatory neuromuscular control strategies to negotiate both the lower and higher contrast obstacles. However, there are some important limitations in interpreting this finding. Previous studies suggest birds are able to detect contrast with thresholds between approximately 15% and 30% (Ghim and Hodos, 2006); however, birds exhibit substantial species-specific variation in visual function (Ghim and Hodos, 2006; Martin, 2011, 2014). To our knowledge, guinea fowl visual function has not been studied in detail. If guinea fowl possess high visual acuity and contrast sensitivity, the obstacle contrast manipulation used here may not have introduced adequate perceptual change to influence anticipatory control. Even in the low-contrast condition, the obstacles may have been visible as a result of shadowing effects. In addition, the treadmill environment may present an ecologically unnatural condition that affects how terrain is perceived, because much of the visual field is static and contradictory to the moving treadmill surface (Prokop et al., 1997). Nonetheless, our finding of significant anticipatory changes at the slower speed in both high- and low-contrast conditions suggests that guinea fowl do perceive sufficient visual information at both contrast conditions, at least when obstacle viewing time is sufficient.

Consistent with hypothesis 2, we observed greater anticipatory muscle modulation (preceding obstacle contact) at the slower treadmill speed. Although guinea fowl exhibited anticipatory increases in muscle activity in advance of obstacles at both speeds, these shifts were larger in magnitude (Fig. 8) and spanned more hindlimb muscles (six versus two; Fig. 5) at the slower speed. This suggests that guinea fowl increase anticipatory muscle recruitment when given more time to visually assess oncoming terrain. The results are consistent with the time dependency of anticipatory control, probably due to delays associated with visuomotor modulation via descending pathways (Patla et al., 1991). Our study provides evidence that, when timely visual sensory information is available, birds do adjust muscle recruitment in anticipation of terrain changes on a treadmill.

### Interpretation of possible underlying mechanisms for observed speed effects

Considering available evidence across overground and treadmill obstacle negotiation studies, we suggest that the time available for visuomotor processing may be more critical than movement speed per se in determining whether birds use anticipatory strategies. When running overground, birds use anticipatory changes in leg and body dynamics to achieve steady gait on the obstacle, even at fast running speeds (Birn-Jeffery, 2012; Birn-Jeffery and Daley, 2012; Birn-Jeffery et al., 2014). This may be because animals can adjust their gaze distance with speed to allow for visuomotor latencies when running overground. In treadmill conditions, gaze distances are restricted by the length of the treadmill. We observed more substantial anticipatory neuromuscular changes during slower speed treadmill obstacle negotiation, which provides longer obstacle viewing time. While we did observe some significant increases in muscle activity (*E*_tot_) before obstacle contact at the higher speed (two out of eight muscles; Fig. 5), we did not observe significant anticipatory shifts in kinematic timing (Fig. 8). In contrast, overground studies found anticipatory shifts in kinematics across speeds (Birn-Jeffery, 2012; Birn-Jeffery and Daley, 2012; Birn-Jeffery et al., 2014). On the treadmill, the maximum time between obstacle appearance and encounter was approximately one stride period, whereas overground, the birds could view obstacles at least two strides in advance (Birn-Jeffery, 2012; Birn-Jeffery and Daley, 2012; Birn-Jeffery et al., 2014). We hypothesize that obstacle viewing time (the time between the visual cue and obstacle contact) is a key factor in the ability to make visuomotor adjustments for altered terrain. However, future experiments that vary obstacle viewing time independent of locomotor speed will be necessary to directly test this interpretation.

We also observed differences between treadmill speeds in the timing of reactionary muscle modulation (after obstacle contact), which may be related to spinal reflex feedback latencies. While short-latency spinal reflexes contribute substantially to motor output at slow and fast speeds, reflexes are highly modulated depending on task (Capaday and Stein, 1987; Ferris, 1999; Stein and Capaday, 1988; Zehr and Stein, 1999; Donelan et al., 2009). At slower speeds, short-latency feedback delays are small relative to stride duration (∼10–25%), allowing larger within-stride feedback adjustments in response to a sensed perturbation (Reis, 1961; Cavanagh and Komi, 1979; Duysens and Loeb, 1980). At higher speeds, short-latency feedback delays can be greater than 50% of stance duration, which can make reflexes destabilizing (Kuo, 2002). This may explain why short-latency reflex responses tend to be down-regulated at higher speeds (Capaday and Stein, 1987; Ferris, 1999). Comparing the two speed conditions in the current study, the slower speed showed higher positive scores for PC1 on the obstacle stride (S0; Fig. 8). In contrast, the faster speed showed higher positive scores for PC1 in the obstacle dismount stride (CL+1; Fig. 8). Thus, the slower speed showed greater within-stride modulation of activity upon obstacle contact, whereas the higher speed relied more heavily on recovery upon dismount from the obstacle. These findings are consistent with a greater reliance on longer latency responses at higher speeds, similar to previous studies (da Silva et al., 2011).

Our findings are also consistent with a shift in intrinsic mechanical stability between speed conditions. We define intrinsic stability mechanisms as those that arise from the natural dynamics of the mechanical system due to inertia, momentum and mechanical energy of the body and limbs (Jindrich and Full, 2002; Moritz and Farley, 2004; Matthis and Fajen, 2013). Despite substantial and widespread shifts in muscle recruitment during obstacle negotiation at both speeds, we observed little change in kinematic timing at the higher speed (Fig. 9B), with a significant change only in the swing duration of the obstacle dismount stride (CL+1). In contrast, the slower speed showed significant shifts in kinematic timing in strides before, during and following obstacle contact (Fig. 9A). The mechanical effects of altered recruitment may be restricted at higher speed as a result of the combined effects of neuromechanical delays and increased intrinsic stability. Intrinsic mechanical stability can be beneficial in bridging unavoidable neural control gaps (Daley et al., 2009; John et al., 2013). However, such mechanical effects are necessarily bi-directional – while intrinsic stability reduces sensitivity to external perturbations, it also reduces responsiveness to changes in muscle activity. Previous studies in cockroaches found that the effects of a specific increase in muscle activation on body dynamics depend strongly on dynamic context (Sponberg et al., 2011a,b). Thus, at different speeds, similar increases in EMG activity may not result in similar mechanical effects. In the current results, the absence of kinematic shifts at the higher speeds, even in strides with significantly increased muscle activity (e.g. strides CL−1 and S0; Figs 5, 9), suggests greater intrinsic stability at the higher speed.

Changing treadmill speed induces other integrated speed effects, including a shift in stance duration, duty factor, peak forces and gait dynamics. Avian bipeds exhibit a gradual transition between walking and running, including ‘grounded running’ at intermediate speeds (Gatesy and Biewener, 1991). This makes it difficult to clearly distinguish between gaits without ground reaction forces and detailed body dynamics, which we do not have here. Nonetheless, higher speed does require larger peak forces and higher muscle activation to support body weight. To focus our analysis specifically on obstacle negotiation strategy, we normalized *E*_tot_ relative to the level terrain mean at the same speed, so that pair-wise comparisons between stride categories reflected shifts during obstacle negotiation, not shifts associated with speed alone. Additionally, we used statistical models that included speed as an independent factor and a speed×stride ID interaction term, which allowed quantification of generic speed effects separate from speed-specific obstacle negotiation strategy (see Results, ‘Statistical summary’). Further, the PCA results revealed similar co-variance patterns among hindlimb muscles between speeds. These findings suggest similar overall neuromuscular control for locomotion between speeds, despite some shifts in the use of anticipatory, reactionary and intrinsic stability mechanisms, discussed above.

### Implications for neuromechanical control models of bipedal locomotion

Previous work has suggested a proximo-distal gradient in limb neuromuscular function, in which distal limb muscles exhibit greater reactionary modulation due to reflex feedback and intrinsic mechanical sensitivity (Daley et al., 2007). In the current study, however, we did not find evidence for a proximo-distal gradient. This is particularly evident from the PCA, which showed that recruitment co-varied strongly across many hindlimb muscles (Fig. 8), without a proximo-distal distinction.

Why was there no proximo-distal gradient, despite its previous observation at the level of limb joint mechanics? In the current study, both anticipatory, feedforward changes and feedback-mediated changes are likely to have contributed to the observed changes in recruitment. In contrast, the previous study (Daley et al., 2007) focused on the ‘reactive’ response to an unexpected perturbation and not an anticipated manoeuvre. The observed proximo-distal gradient is likely to have resulted from a combination of intrinsic mechanical factors and feedback-mediated changes, without anticipatory effects. Here, we did observe slightly higher magnitude shifts in EMG activity in the distal compared with the proximal muscles in the obstacle dismount stride (Fig. 5; CL+1), consistent with higher gain load-dependent feedback in the distal muscles, as suggested in a previous cat study (Nichols and Ross, 2009). However, an important limitation of the current and previous guinea fowl studies is the lack of simultaneous measurements of joint dynamics and hindlimb muscle activity, as the current study focused on muscle activity, whereas the previous one focused on joint dynamics (Daley et al., 2007). The link between muscle activation and joint dynamics is indirect, depending on the physical properties of the limb segments and the action of multi-articular muscles in transmitting force and energy between joints (Prilutsky, 2000). It therefore remains to be investigated whether the limb-wide co-variation in muscle recruitment observed here maps to comparable limb-wide changes in joint dynamics.

Nonetheless, the PCA results here do reveal synergistic co-activation of muscles across the limb, rather than independent control of individual muscles. This finding suggests that a relatively simple reduced-order control model might be able to reproduce the observed limb-wide co-variation in muscle activity, consistent with the idea that control is simplified through muscle synergies arising from spinal neural networks (d'Avella and Bizzi, 2005; Chvatal and Ting, 2012; Bizzi and Cheung, 2013). For example, control commands might relate to limb extension and limb retraction, representing a reduced-order model of bipedal locomotion similar to those presented in Birn-Jeffery et al. (2014) and Van Why et al. (2014). In future work, it will be interesting to investigate the specific mapping between detailed musculoskeletal dynamics and reduced-order neuromechanical control ‘templates’ (*sensu* Full and Koditschek, 1999) for bipedal locomotion.

### Conclusions

Guinea fowl make greater use of anticipatory control strategies during slower speed treadmill obstacle negotiation, compared with higher speed, demonstrating context-dependent neuromuscular control. We suggest that this finding relates to the greater time available for visuomotor processing at slower speeds on a treadmill, due to higher available obstacle viewing time. When taken in the context of previous literature, our results suggest that a treadmill environment may enhance speed-dependent differences in sensorimotor control, possibly due to both sensory and mechanical effects, including restricted visual information, restricted manoeuvring space on the treadmill belt, and speed-related changes in intrinsic mechanical stability.

## MATERIALS AND METHODS

### Animals and training

We obtained six adult guinea fowl, *Numida meleagris* (Linnaeus 1758) with a body mass of 1.6±0.23 kg from Devon, UK. We trained birds to run on a level motorized treadmill (Woodway, Waukesha, WI, USA) at speeds up to 2 m s^−1^, with training sessions of 15–20 min in duration, with breaks for 2 min as needed. Each bird received 3–4 days training per week for 3 weeks before our study commenced. All experiments were conducted at the RVC Structure and Motion Laboratory under a project licence approved by the college’s Ethics and Welfare committee and granted by the UK Home Office.

### Surgical procedures

Birds received a premedication of 0.2 mg kg^−1^ intramuscular Butorphanol 15 min prior to induction. Sevofluorane was used to induce anaesthesia through a mask, followed by intubation with a non-cuffed endotracheal tube and continued gaseous maintenance of mid-plane anaesthesia throughout the remainder of the procedure. Perioperative antibiotics and anti-inflammatories were administered intramuscularly after induction. Three skin incisions of 3–5 cm were made over the right caudal thigh, cranial thigh and lateral shank to enable direct visualization and intramuscular electrode placement in eight superficially accessible muscles distributed proximal and distal to the knee (Fig. 1). Electrodes had been previously constructed from two strands of 38 gauge Teflon-coated stainless steel (AS 632, Cooner Wire Co., CA, USA) with a staggered 1 mm exposed wire region spaced 1.5 mm apart. The two electrodes were placed simultaneously using sew-through methods and silicon anchors (3×3×2 mm) positioned with a single square knot at the muscle surface–electrode interface (Deban and Carrier, 2002). Wires were tunnelled together subcutaneously through silicon tubing using a looped guide wire through a 1.5 cm incision made over the dorsal synsacrum. Leg incisions were then closed with two metric nylon sutures. The dorsal incision was closed using a purse-string nylon 2 metric suture around the silicon tubing before a nylon finger-trap was secured. All electrodes were then soldered into a D-type multi-pin connector. Excess wiring was re-introduced into the silicon tubing and quick-drying adhesive (Araldite™ Rapid) used to create a protective insulated seal encompassing the tube end and newly soldered connections. All birds recovered to standing within 30 min of surgery. Carprofen was administered at 1 mg kg^−1^ once daily and Enrofloxacin at 10 mg kg^−1^ twice daily during the experimental period. After the data collection was complete, a second anaesthesia was performed as above to enable electrode inspection, verification and removal. All birds recovered and healed post-surgery.

### EMG recordings

The micro-connector on the dorsal synsacrum was connected via a purpose-built lightweight shielded cable to 8 GRASS pre-amplifiers (P511, Natus Neurology Incorporated). EMG signals remained at a constant amplification throughout data collection with low-pass (10 Hz) and high-pass (3 kHz) filtering. EMG signals were sampled using an A/D converter at 4920 Hz using a customized LabView program interface (National Instruments Corporation Ltd, UK).

### Kinematics

Digital high-speed video was recorded in lateral view at 120 Hz (AOS High Resolution). Major joints were highlighted using high-contrast adhesive markers.

### Experimental protocol

The guinea fowl were run for 30 s trials spaced with 10 min rest periods during which the birds were given access to food and water. Three trials were recorded for each condition over a 2 day period. We recorded data at two speeds (0.7 m s^−1^, Froude 0.25; and 1.3 m s^−1^, Froude 0.86) and three terrain conditions at each speed: (i) level, (ii) repeated 5 cm low-contrast obstacles (black), and (iii) repeated 5 cm high-contrast obstacles (black with white stripes). We selected the specific speeds to provide a substantial difference in the obstacle viewing time, while remaining within the range of speeds the birds could comfortably maintain on the treadmill. The treadmill belt was slatted black rubber-coated steel with a 55.8×172.7 cm running surface with sufficient clearance to allow free passage of obstacles beneath the treadmill. Obstacles were constructed from a balsa wood base covered with black neoprene, and high contrast was introduced using white felt stripes. A heavy-duty hook and loop fastener was used to attach the obstacles to the treadmill surface. Four obstacles were placed on sequential slats, creating a 20 cm^2^ obstacle surface to be negotiated once per belt rotation. Birds encountered obstacles every 4–5 strides at the faster speed and every 5–6 strides at the slower speed (because of the shorter stride lengths at the slower speed).

We designed the high- and low-contrast obstacles to maximize the difference in contrast signal between conditions. Birds have lower contrast sensitivity than mammals; however, they are able to detect contrast with thresholds between approximately 15% and 30%, depending on the species and the spatial frequency of the presented contrast signal (Ghim and Hodos, 2006). The obstacles in this study subtended a relatively large visual angle while moving backwards on the treadmill belt, facilitating contrast detection (Ghim and Hodos, 2006). The ‘low-contrast’ obstacles exhibited <10% contrast from the belt (black/black), and the ‘high-contrast’ obstacles exhibited >90% contrast (black/white). Thus, the low-contrast obstacles were near the undetectable range for birds, whereas the high-contrast obstacles were safely within the detectable range, assuming that guinea fowl have contrast sensitivity comparable to that of other birds.

### Data processing

Videos were observed and frame times manually recorded for right-foot stride sequences using Virtual Dub software. Strides were identified based on their sequence in relation to obstacle contact, and as only single-limb instrumentation was undertaken, two possible alternative obstacle negotiation sequences were separately analysed (Fig. 3). A sequentially ordered sequence of strides IDs was re-constructed from these two sequences, representing the full bilateral obstacle negotiation sequence, assuming symmetry between right and left legs. These stride IDs were coded as a fixed effect factor for further analysis and statistics.

Raw EMG signals were used to calculate the myoelectric intensity of the EMG signal in time–frequency space using wavelet decomposition (von Tscharner, 2000; Wakeling et al., 2002). We used a bank of 16 wavelets with time and frequency resolution optimized for muscle, with wavelet centre frequencies ranging from 6.9 to 804.2 Hz (von Tscharner, 2000). The intensity over wavelets 92.4 to 804.2 Hz at each time point was then summed to calculate the instantaneous myoelectric intensity (mV^2^). This provides a smooth trace of EMG intensity over time that accounts for the entire physiological frequency range and acts to exclude noise from the calculation. Instantaneous intensity traces (mV^2^) were cut into strides and categorized by stride, normalized by the mean peak intensity of level terrain strides, for each specific muscle and bird at the same speed. Total myoelectric intensity per stride was calculated by integrating this intensity wave over time (mV^2^ s) for each stride interval. The resulting total intensities (*E*_tot_) were normalized by the level terrain mean at the same speed, prior to further statistical analysis. Such normalization ensured that shifts in *E*_tot_ during obstacle negotiation reflected differences relative to level terrain at the same speed, to minimize effects due to speed alone. Data processing was completed using MATLAB (MathWorks, Inc., Natick, MA, USA).

### Statistics

A linear mixed effects model was used to test for significant effects of speed, contrast (obstacle contrast) and stride ID (obstacle negotiation strategy), on the dependent variables *E*_tot_ for each muscle, stride duration, stance duration and swing duration (Tables 2, 3). Several linear mixed effects models were evaluated in comparison to a reference model, as detailed below. The final model reported is that which resulted in the lowest AIC for all dependent factors, calculated as AIC=2*k*−2log(*L*) (Akaike, 1976), where *k* is the number of predictors in the model and *L* is the maximum likelihood value. The AIC provides a method of comparing the goodness of fit of multiple models, penalizing those with a higher number of parameters, promoting parsimonious model selection. The model with the lowest AIC is preferred. Fixed effects included in model comparison were speed, stride ID, speed×stride ID (to test for the effect of speed on obstacle negotiation strategy), contrast and contrast×stride ID (to test for the effect of contrast on obstacle negotiation strategy). All models included individual (bird ID) as a random effect to account for individual variation. The LME and *post hoc* pairwise comparison Tukey's tests were applied using the open source R software (lme4 and multcomp packages; R Development Core Team, 2008).

Several specific models were evaluated in comparison to a reference model to test the proposed hypotheses and ensure that variance in data was characterized using the simplest possible model. Reference and alternative models were compared, as below, for all dependent factors (where ‘factor’ is one of the dependent factors: *E*_tot_, stride duration, swing duration, stance duration). The reference model includes the independent factors of speed and stride ID, plus the random effect of individual. This model represents the null hypotheses that (1) there is no significant effect of speed on obstacle negotiation strategy (omitting the speed×stride ID term) and (2) there is no significant effect of contrast on obstacle negotiation strategy (omitting the contrast×stride ID term):



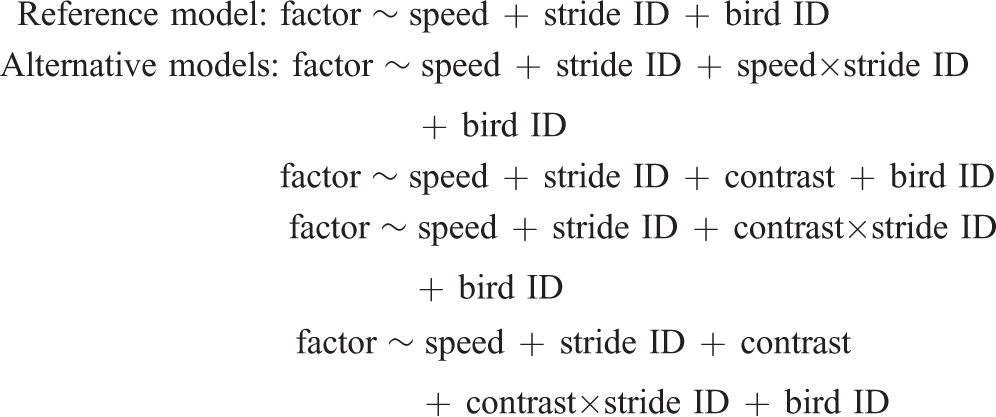


The model reported with the lowest AIC for each factor was:

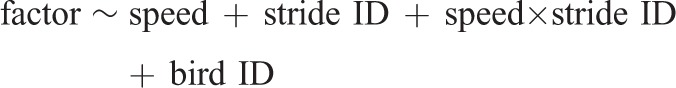


We used Tukey’s *post hoc* pairwise comparisons to further explore the specific changes in obstacle negotiation, comparing each obstacle negotiation stride with reference mid-flat strides in low-contrast terrain, within each speed (significance value set at *P*≤0.05).

A PCA was performed in MATLAB (MathWorks, Inc.) to analyse covariance patterns in *E*_tot_ across all eight measured hindlimb muscles, with the PCA dataset including the grand mean *E*_tot_ for each muscle and each stride category across both speeds and all three terrains (level terrain, high-contrast obstacles, low-contrast obstacles).
